# DPP-4 inhibitor induces FGF21 expression via sirtuin 1 signaling and improves myocardial energy metabolism

**DOI:** 10.1007/s00380-020-01711-z

**Published:** 2020-10-18

**Authors:** Nozomi Furukawa, Norimichi Koitabashi, Hiroki Matsui, Hiroaki Sunaga, Yogi Umbarawan, Mas Rizky A. A. Syamsunarno, Aiko Yamaguchi, Masaru Obokata, Hirofumi Hanaoka, Tomoyuki Yokoyama, Masahiko Kurabayashi

**Affiliations:** 1 Department of Cardiovascular Medicine, Gunma University Graduate School of Medicine, 3-39-22, Showa-machi, Maebashi, Gunma 371-8511 Japan; 2 Department of Laboratory Sciences, Gunma University Graduate School of Health Sciences, Maebashi, Gunma Japan; 3 Department of Bioimaging Information Analysis, Gunma University Graduate School of Medicine, Maebashi, Gunma Japan

**Keywords:** Heart failure, Hypertrophy, Metabolism, Cardiac fibroblast

## Abstract

Dipeptidyl peptidase-4 (DPP-4) inhibitors are widely used incretin-based therapy for the treatment of type 2 diabetes. We investigated the cardioprotective effect of a DPP-4 inhibitor, vildagliptin (*vilda*), on myocardial metabolism and cardiac performance under pressure overload. Mice were treated with either vehicle or *vilda*, followed by transverse aortic constriction (TAC). After 3 weeks of TAC, cardiac hypertrophy and impairment of systolic function were attenuated in *vilda*-treated mice. Pressure–volume analysis showed that *vilda* treatment significantly improved left-ventricular contractile efficiency in TAC heart. Myocardial energy substrate analysis showed that *vilda* treatment significantly increased glucose uptake as well as fatty acid uptake. Fibroblast growth factor 21 (FGF21), a peptide involved in the regulation of energy metabolism, increased in TAC heart and was further increased by *vilda* treatment. FGF21 was strongly expressed in cardiac fibroblasts than in cardiomyocytes in mouse heart after TAC with *vilda* treatment. *Vilda* treatment markedly induced FGF21 expression in human cardiac fibroblasts through a sirtuin (Sirt) 1-mediated pathway, suggesting that fibroblast-mediated FGF21 expression may regulate energy metabolism and exert *vilda*-mediated beneficial effects in stressed heart. *Vilda* induced a metabolic regulator, FGF21 expression in cardiac fibroblasts via Sirt1, and increased contractile efficiency in murine pressure-overloaded heart.

## Introduction

Heart failure (HF) is caused by various heart diseases including cardiomyopathy, hypertensive heart disease, valvular disease, and ischemic heart disease. Chronic HF is commonly related to lifestyle diseases like obesity, hypertension, dyslipidemia, and diabetes mellitus [1–3]. In the Framingham Heart Study, the risk of heart failure in patients with diabetes was increased twofold in males and fivefold in females compared to patients without diabetes [4]. Dipeptidyl peptidase-4 (DPP-4) inhibitors are a class of oral hypoglycemics that block the enzyme DPP-4. DPP-4 is a serine protease, which is expressed in many cells and tissues throughout the body [5]. DPP-4 regulates pathophysiological function through degrading many biologically active substances [5]. Experimental studies have reported that a DPP-4 inhibitor attenuated left-ventricular dysfunction of type 1 diabetic rats produced by streptozocin injection [6]. Moreover, vildagliptin (*vilda*) improved cardiac hypertrophy and left-ventricular dysfunction in hypertrophied mice following aortic constriction [7]. However, the protective effect of DPP-4 inhibitors on heart function remains controversial [8]. Several small pilot trials showed the beneficial effects of DPP4 inhibitors on cardiac function and remodeling in patients with diabetes [9, 10]. Interestingly, it has been shown that DPP-4 inhibitor treatment improved glucose uptake in nonischemic cardiomyopathy [11]. In addition, a dobutamine stress echocardiogram study showed that a DPP-4 inhibitor improved myocardial responses in ischemic myocardium [12]. These results suggest that DPP-4 inhibitors might have a potent beneficial effect on energetic responses in damaged myocardium.

In this study, we examined the anti-hypertrophic and cardioprotective effects of a DPP-4 inhibitor, *vilda*, in a murine pressure-overloaded model under non-diabetic conditions. Since *vilda* showed a cardioprotective effect through cardiac energy efficiency, we focused on a peptide hormone, fibroblast growth factor 21 (FGF21), which has been shown to regulate energy homeostasis in several organs [13]. We showed that FGF21 protein expression was increased in pressure-overloaded mouse heart compared to control heart, and was further increased by *vilda* treatment. *Vilda* increased cardiac-fibroblast FGF21 in vitro. Furthermore, FGF21 expression was associated with an increase in sirtuin (Sirt) 1 expression. These results suggest that cardiac-fibroblast-mediated FGF21 expression may regulate energy metabolism in stressed heart, and that the DPP-4 inhibitor *vilda* enhances this pathway.

## Materials and methods

### Reagents

The DPP-4 inhibitor vildagliptin (*vilda*) used in this study was provided by Novartis Pharma AG (Basel, Switzerland). For in vivo studies, we used a dose of 50 mg/kg/day of *vilda* [14, 15]. *Vilda* was given to mice by oral administration using a feeding needle for 4 weeks, beginning at 17 weeks of age. After 1 week of *vilda* treatment, transverse aortic constriction (TAC) surgery was performed. For in vitro studies using cultured cells, we used concentrations of 10 nM of *vilda*. Resveratrol (Sirt1 activator) was purchased from Sigma-Aldrich (Saint Louis, MO, USA).

### Animal model

All animal experiments were performed in accordance with protocols approved by the Committee of Experimental Animal Research of Gunma University (Permit Number; 11-073). All animal models and experimental procedures conformed to the NIH Guide for the Care and Use of Laboratory Animals. 4 week male C57BL/6j mice were purchased from Japan SLC inc. Japan. Pressure overload was produced by constricting the transverse aorta as previously described [16]. The aorta was approached via a minimal sternal incision and a 7–0 ligature was placed around the vessel using a 26G needle to ensure consistent occlusion. Sham-operated mice underwent the same surgery without constriction.

### Echocardiography

In vivo cardiac morphology was assessed by trans-thoracic echocardiography (EUB-7500; Hitachi Medical Systems, Tokyo, Japan) in conscious mice. M-mode left ventricular (LV) end-systolic and end-diastolic dimensions were averaged from 3 to 5 beats. LV percent fractional shortening and mass were calculated as described [16]. The studies and analysis were performed blinded as to the experimental groups.

### In vivo hemodynamics

In vivo LV function was assessed using a pressure–volume (PV) conductance catheter as described previously [16, 17]. Mice were anesthetized, intubated, and ventilated. The LV apex was exposed between the seventh and eighth ribs, and a 1.2-Fr PV conductance catheter (Transonic Systems Inc, Ithaca, NY, USA) was advanced through the apex to lie along the longitudinal axis. Data were assessed at steady state and during preload reduction. Data were digitized and analyzed with the ADVantage PV system (ADV500; Transonic Systems Inc).

### Biodistribution of [15-(*p*-iodophenyl)-3-(*R*, *S*)-methyl pentadecanoic acid] and 2-fluorodeoxyglucose

Cardiac uptake and biodistribution of 15-(*p*-iodophenyl)-3-(*R*, *S*)-methyl pentadecanoic acid (^125^I-BMIPP) and 2-fluorodeoxyglucose (^18^F-FDG) were determined as described previously [18–20].

### Tissue preparation

Following induction of deep anesthesia with isoflurane, mice were sacrificed with 5% potassium chloride perfusion to arrest the heart in diastole and rinse out residual blood, excised immediately. The tissue was frozen rapidly and stored at − 80 °C until used in western blot and real-time PCR analysis. The hearts were inflated by instilling with 10% formalin and fixed for 24 h before paraffin embedding. Individual serial sections (2 μm) were prepared for histological analysis. Fibrosis and capillary density were estimated as described previously [16].

### Immunostaining and confocal microscopy

Immuno-fluorescent staining and analysis were performed as previously described [16]. Fluorescence immunohistochemistry was performed as follows. Paraffin embedded sections were stained with rabbit monoclonal FGF21 (Abcam, ab171941, Cambridge, UK) using an Alexa Fluor 488-conjugated anti-rabbit TSA kit (Thermo Fisher Scientific, Waltham, MA, USA) according to the manufacturer’s protocol. Mouse anti-tropomyosin antibody (GTX17784: GeneTex, Irvine, CA, USA) and Alexa Fluor 546 goat anti-mouse IgG (Thermo Fisher Scientific) were used for cardiomyocyte immunostaining. WGA (Alexa Fluor 637; Thermo Fisher Scientific) was used for counterstaining of the plasma membrane/extracellular matrix. Confocal analysis was performed on Zeiss LSM510 and LSM880 laser scanning confocal microscopes [16].

### Culture of human cardiac-fibroblast (HCF) cells

HCF cells were obtained from Cell Systems (Kirkland, WA, USA). HCF cells were cultured using Fibroblast Media2 (Cell Systems) supplemented with 2% fetal calf serum, 1% penicillin/streptomycin, 1 ng/mL Basic Fibroblast Growth Factor, and 5 μg/mL insulin in a 5% CO_2_ atmosphere at 37 °C. HCF cells were used between passages 3 and 5 in all experiments. Serum-starved HCF cells were incubated in the presence of 0.1% Insulin-Transferrin-Selenium-X (Thermo Fischer Scientific).

### Gene silencing with siRNA

siRNA oligonucleotides were purchased from BioNEER (Daejeon, Korea). Construction of siRNA oligonucleotides were as follows: human siDPP4: CUU UUG AAC AGA GUA AAU; human siSirt1: CCA GAG CCC AAG CCA ATT, BioNEER (Daejeon, Republic of Korea). SiRNA was transfected with Lipofectamine RNAiMAX Reagent (Thermo Fisher Scientific) according to the manufacturer’s protocol.

### RNA isolation and real-time RT-PCR

Total RNA was extracted from the mouse heart tissue using ISOGEN reagent (Takara Bio, Kyoto, Japan) according to the manufacturer’s protocol. One microgram of RNA was used for reverse transcription with the RNA LA PCR Kit (Takara Bio), and qRT-PCR analysis was performed using THUNDERBIRD SYBR qPCR Mix (TOYOBO, Osaka, Japan) according to the manufacturers’ protocol. qRT-PCR analysis was performed using an MX3000P quantitative system (Stratagene, CA, USA). The primer sequences were as follows: FGF21[forward: CTGGGGGTCTACCAAGCATA, reverse: GTCCTCCAGCAGCAGTTCTC], GAPDH[forward: AGCCCCCAGTCTGTATCCTT, reverse: TCCACCACCCTGTTGCTGTA].

### Western blot analysis

Tissue samples and cells were homogenized on ice in RIPA buffer (20 mM Tris–HCl (pH 7.4), 150 mM NaCl, 1% NP-40, 1% sodium deoxycholate, and 0.1% SDS) containing complete mini and phos-STOP solution (Roche, Basel, Switzerland). The mixture was centrifuged at 15,000 rpm for 30 min and the supernatant was subjected to SDS-PAGE. Protein concentrations were determined by the Bradford method using a colorimetric assay (Bio-Rad, Hercules, CA, USA). Western blot analysis was performed according to standard procedures using the following primary antibodies: rabbit monoclonal FGF21 (Abcam, ab171941, 1:250), and from Cell Signaling Technology (Danvers, MA, USA), GAPDH (#2118, 1:500), Sirt1(#9475, 1:250), DPP4/CD26 (#67138S, 1:250), phospho-AMP-activated protein kinase (AMPK) α (Thr172; #2535, 1:250), AMPK (#2603, 1:250), Phospho-FoxO3a (S253; #9466S, 1:250), and FoxO3a (#2497S, 1:500). Antigens were detected using Immobilon Western HRP Substrate (Millipore, Billerica, MA, USA) after incubation with horseradish peroxidase conjugated anti-rabbit IgG. The density of bands was quantified using ImageJ software.

### ELISA assay for FGF21

The protein levels of FGF21 in HCF culture supernatant were quantified using an enzyme-linked immunosorbent assay (ELISA) kits for human FGF21 (R&D Systems, Minneapolis, MN, USA), according to the manufacturer’s instructions.

### Statistical analysis

All values are expressed as mean ± SEM. Comparisons between two groups were performed using an unpaired two-tailed Student’s *t* test with Welch’s correction. An unpaired Student’s *t* test was performed for each pair of four groups and subsequent multiple comparisons were made using Bonferroni's method. *P* < 0.05 was considered significant.

## Results

### Vildagliptin treatment significantly attenuated cardiac hypertrophy and dysfunction

**Fig. 1:**
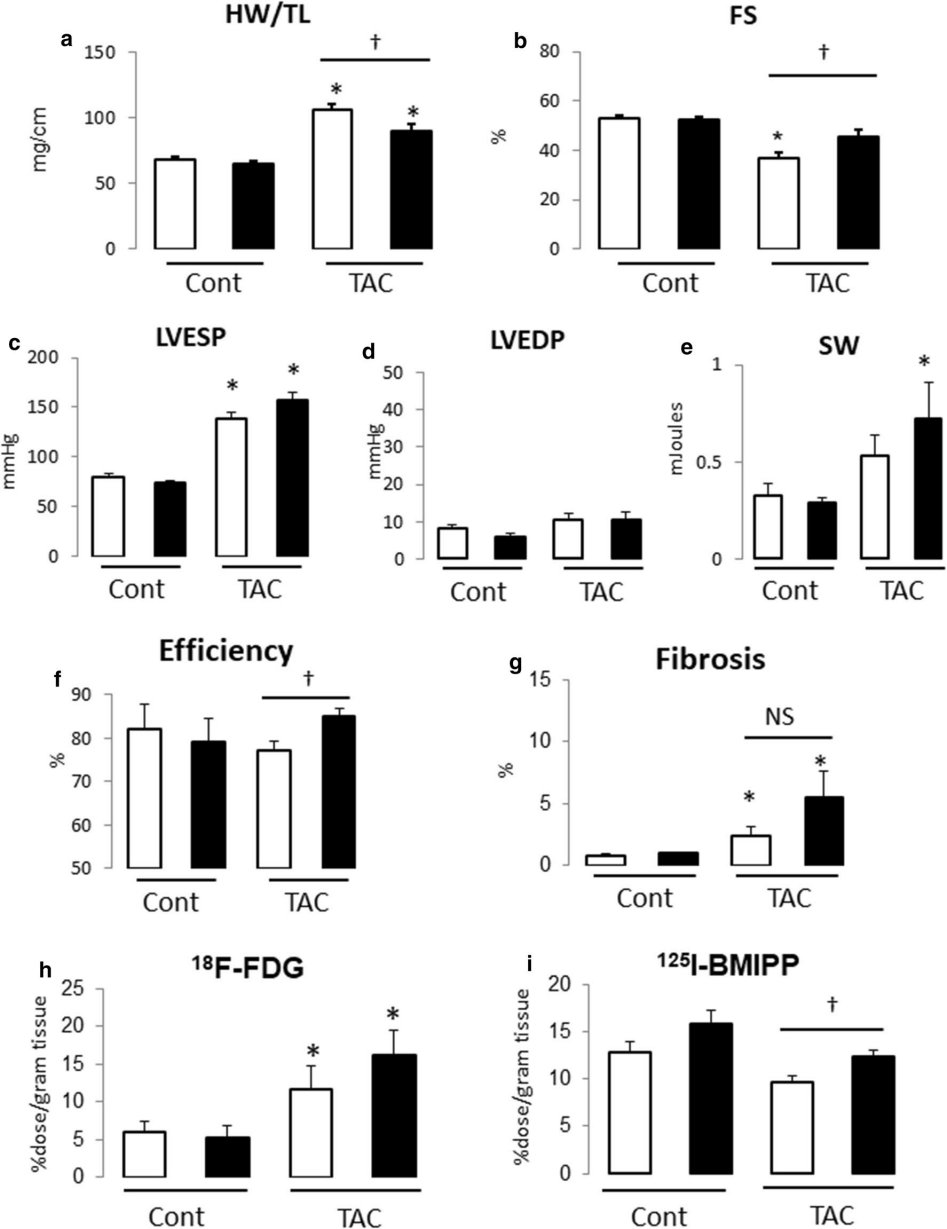
Effect of *vilda* treatment on cardiac hypertrophy and dysfunction in pressure-overloaded non-diabetic mouse heart. **a** Heart weight/tibia length ratio (HW/TL). **b** Fractional shortening (FS) measured by echocardiography. Open bar, vehicle-treated; closed bar, *vilda*-treated (**a**, **b**; Cont with vehicle, *N* = 6; Cont with *vilda*, *N* = 6; TAC with vehicle, *N* = 9; TAC with *Vilda*, *N* = 10). **c**–**f** Conductance catheter measurements (*N* = 5–7 of each group). Open bar, vehicle-treated; closed bar, *vilda*-treated. LVESP, left-ventricular end-systolic pressure (**c**); LVEDP, left-ventricular end-diastolic pressure (**d**); SW, stroke work (**e**); efficiency estimated by SW/pressure–volume area (**f**). **g** Myocardial fibrosis area estimated by Masson’s trichrome staining. **h**, **i** Glucose uptake and free fatty acid (FFA) uptake in hypertrophied heart and *vilda*’s effect. Glucose uptake was estimated by ^18^F-FDG (2-fluorodeoxyglucose) uptake in heart (**h**). FFA uptake was estimated by ^125^I-BMIPP uptake in heart (**i**). Statistical analysis was performed unpaired two-tailed Student’s *t* test with Welch’s correction. *P < 0.05 vs control. ^†^*P* < 0.05 vs vehicle-treated group

We provoke cardiac hypertrophy by transverse aortic constriction (TAC). Treatment with vilda or vehicle was started 1 week before TAC. After 3 weeks of TAC, we measured cardiac function by echocardiography and PV conductance catheter measurement, and examined cardiac energy metabolism by evaluating BMIPP or FDG uptake. TAC developed cardiac hypertrophy (Fig. 1a) and dysfunction (Fig. 1b). These changes were attenuated in the *vilda*-treatment group (Fig. 1a, b).

**Fig. 2:**
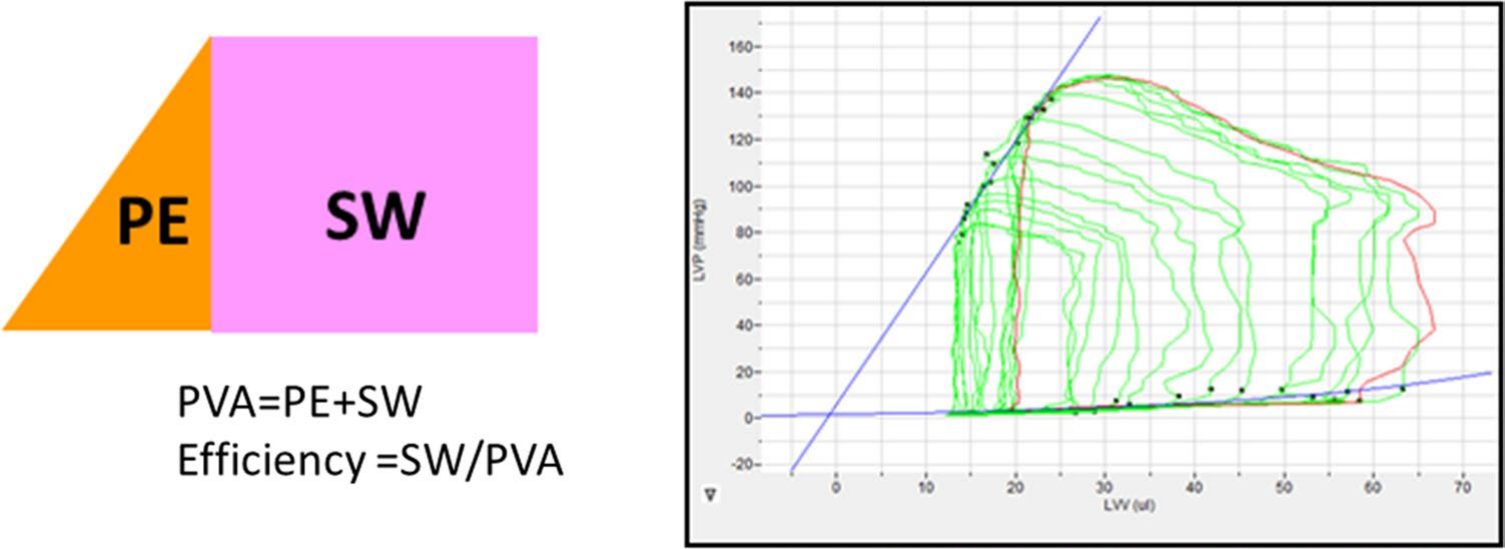
Representative PV loop after TAC

PV catheter measurements (summarized in Table) showed that left-ventricular end-systolic pressure (LVESP) was comparably elevated by TAC in the vehicle- and *vilda*-treated groups (Fig. 1c). LV end-diastolic pressure (LVEDP) showed no significant difference between the two groups (Fig. 1d). *Vilda *treatment did not show any effect on LVESP or LVESP in control mice. Systolic parameters (*dP*/*dt*_max_, Ees, and PRSW) and diastolic parameters (− *dP*/*dt*_min_, Tau and Stiffness) were almost identical between vehicle and *vilda*-treated TAC groups (Table 1). PV loop analysis can estimate several parameters for left-ventricular energetic performance (Fig. 2). Pressure–volume area (PVA) is a parameter describing energy consumption in the working heart. Stroke work (SW) after TAC was significantly increased (Fig. 1e) and efficiency (SW/PVA) was improved in the *vilda*-treatment group (Fig. 1f). Myocardial fibrosis estimated with Masson’s trichrome staining showed significant increases by TAC but no significant difference between the two treatment groups (Fig. 1g).

**Table 1:** Pressure–volume analysis parameters

Parameters	Cont		TAC	
	Vehicle	Vildagliptin	Vehicle	Vildagliptin
Heart rate (/min)	544 ± 22	516 ± 58	518 ± 18	493 ± 15
LVESP (mmHg)	80.0 ± 3.6	73.4 ± 2.4	138.1 ± 6.5*	156.9 ± 7.8*
LVEDP (mmHg)	8.2 ± 0.8	5.9 ± 0.8	10.7 ± 1.7	10.5 ± 2.0
*dP*/*dt*_max_ (mmHg/s)	10,894 ± 1409	8866 ± 1897	10,663 ± 780	10,600 ± 845
− *dP*/*dt*_min_ (mmHg/s)	− 6661 ± 486	− 5688 ± 439	− 7657 ± 482	− 8133 ± 601
Stroke work (mJ)	0.33 ± 0.06	0.29 ± 0.02	0.53 ± 0.10	0.72 ± 0.19*
Efficiency (%)	82.2 ± 5.5	79.0 ± 5.6	77.3 ± 1.9	85.1 ± 1.5^†^
Tau (ms)	9.4 ± 0.3	9.7 ± 0.6	12.4 ± 0.9*	13.2 ± 1.3*
Ees (mmHg/µL)	15.0 ± 3.2	8.6 ± 3.0	32.0 ± 10.6*	58.7 ± 14.3*
PRSW (mmHg)	76.3 ± 8.9	72.3 ± 4.9	137.8 ± 9.0*	127.4 ± 9.7*
Stiffness	0.04 ± 0.01	0.05 ± 0.02	0.04 ± 0.01	0.04 ± 0.02

*LVESP* left-ventricular end-systolic pressure, *LVEDP* left-ventricular end-diastolic pressure, *dP*/*dt*_max_ peak rate of pressure rise, − *dP*/*dt*_min_ peak rate of pressure decline, *Tau* relaxation time constant calculated by Glantz method, *Ees* end-systolic elastance, *PRSW* preload recruited stroke work, *P < 0.05 vs sham, ^†^*P* < 0.05 vs vehicle-treated TAC

### Vildagliptin treatment increased glucose and FFA uptake in hypertrophied heart in non-diabetic mice

We next examined the effects of *vilda* on myocardial energy metabolism in the heart subjected to TAC in a radioisotope study. FDG (glucose analogue) uptake was significantly increased after TAC, which was further increased in the *vilda*-treatment group (Fig. 1h). In contrast, BMIPP (fatty acid analogue) uptake was decreased by TAC in the vehicle-treatment group, which was blunted in the *vilda*-treatment group (Fig. 1i). Fasting blood glucose was similar between the vehicle- and *vilda*-treatment groups (data not shown), suggesting that the observed difference in FDG and BMIPP uptake between the two groups was independent of blood glucose levels.

### FGF21 expression in cardiac myocytes and stromal cells following pressure overload

**Fig. 3:**
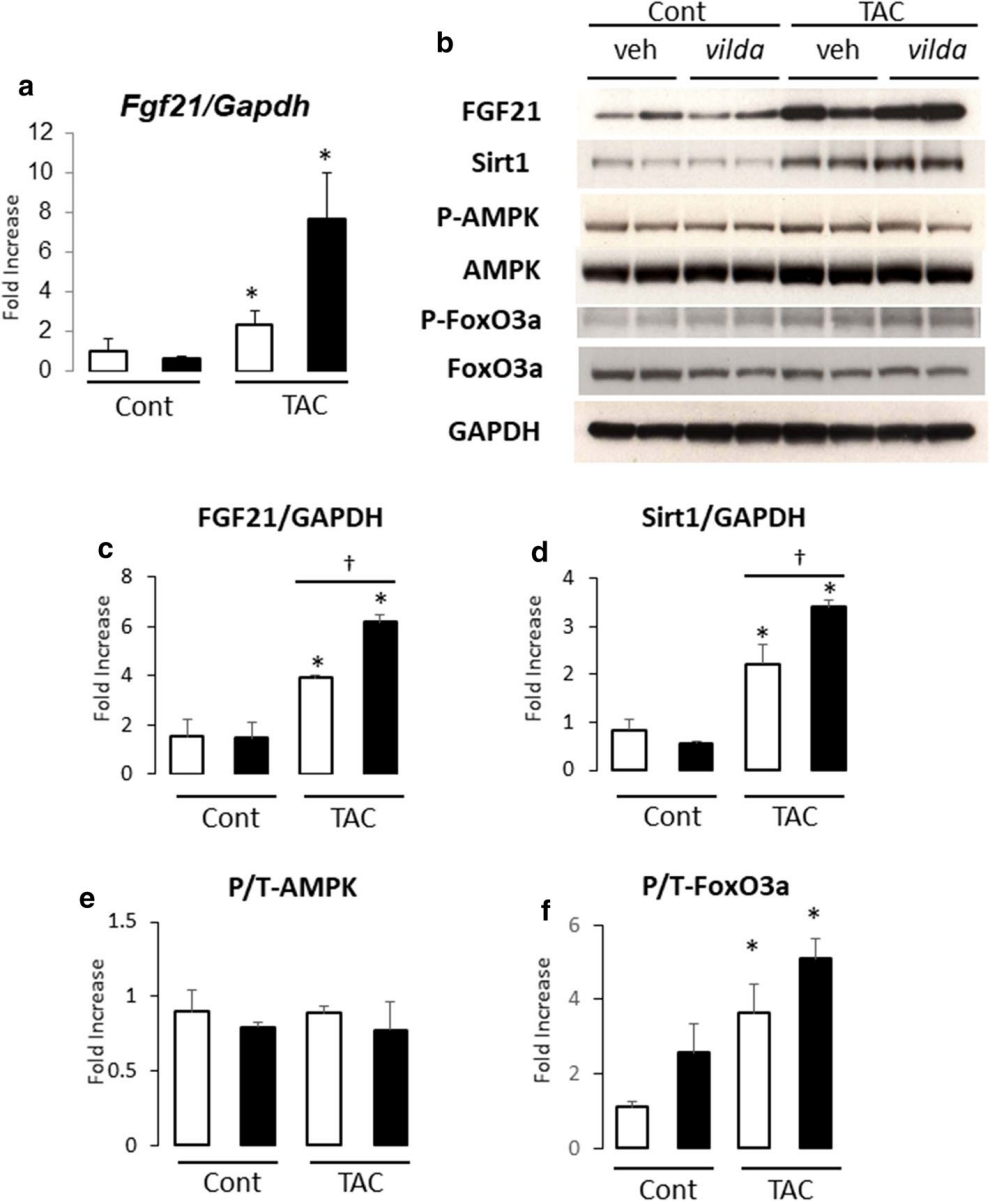
*Vilda* increased cardiac FGF21 expression levels in non-diabetic mouse heart with TAC. Open bar, vehicle-treated; closed bar, *vilda*-treated. **a** Myocardial FGF21 mRNA expression by real-time RT-PCR. **b** Representative western blot of mouse heart. **c**–**f** Summarized protein expression data for FGF21 (**c**), Sirt1 (**d**), phosphorylation levels of AMPK (**e**), and FoxO3a (**f**) estimated by the ratio of phosphorylated protein and total protein intensities. *N* = 4 for each experiment. Statistical analysis was performed unpaired two-tailed Student’s *t* test with Welch’s correction. *P < 0.05 vs control. ^†^*P* < 0.05 vs vehicle-treated group

Fibroblast growth factor 21 (FGF21), which belongs to the FGF family, is an endocrine factor that is secreted mainly by the liver and functions in glucose/lipid homeostasis [21–23]. Multiple studies have shown that cardiac production of FGF21 is up-regulated during cardiac hypertrophy, and is associated with the induction of anti-oxidant gene expression in cardiac hypertrophy and heart failure [24–27]. Therefore, we investigated whether *vilda* induces FGF21 expression in the heart. RNA and protein expression of FGF21 were significantly increased after 3 weeks of TAC (Fig. 3a, b). Myocardial FGF21 expression was further increased with *vilda* treatment (Fig. 3a–c).

To explore the molecular mechanism underlying the prominent induction of FGF21 expression by *vilda*-TAC in the heart, we examined the regulation of FGF21 expression by several signaling molecules including Sirt1, AMPK, and FoxO3a [26–28]. Notably, the expression of Sirt1 was increased in mouse heart following TAC and *vilda*-treatment significantly increased Sirt1 in heart with TAC (Fig. 3b, d). Phosphorylation of AMP-activated protein kinase (AMPK) was not significantly increased in the TAC heart (Fig. 3b, e). Phosphorylation of FoxO3a was increased in the TAC heart but not significantly increased by *vilda *treatment (Fig. 3b, f).

**Fig. 4:**
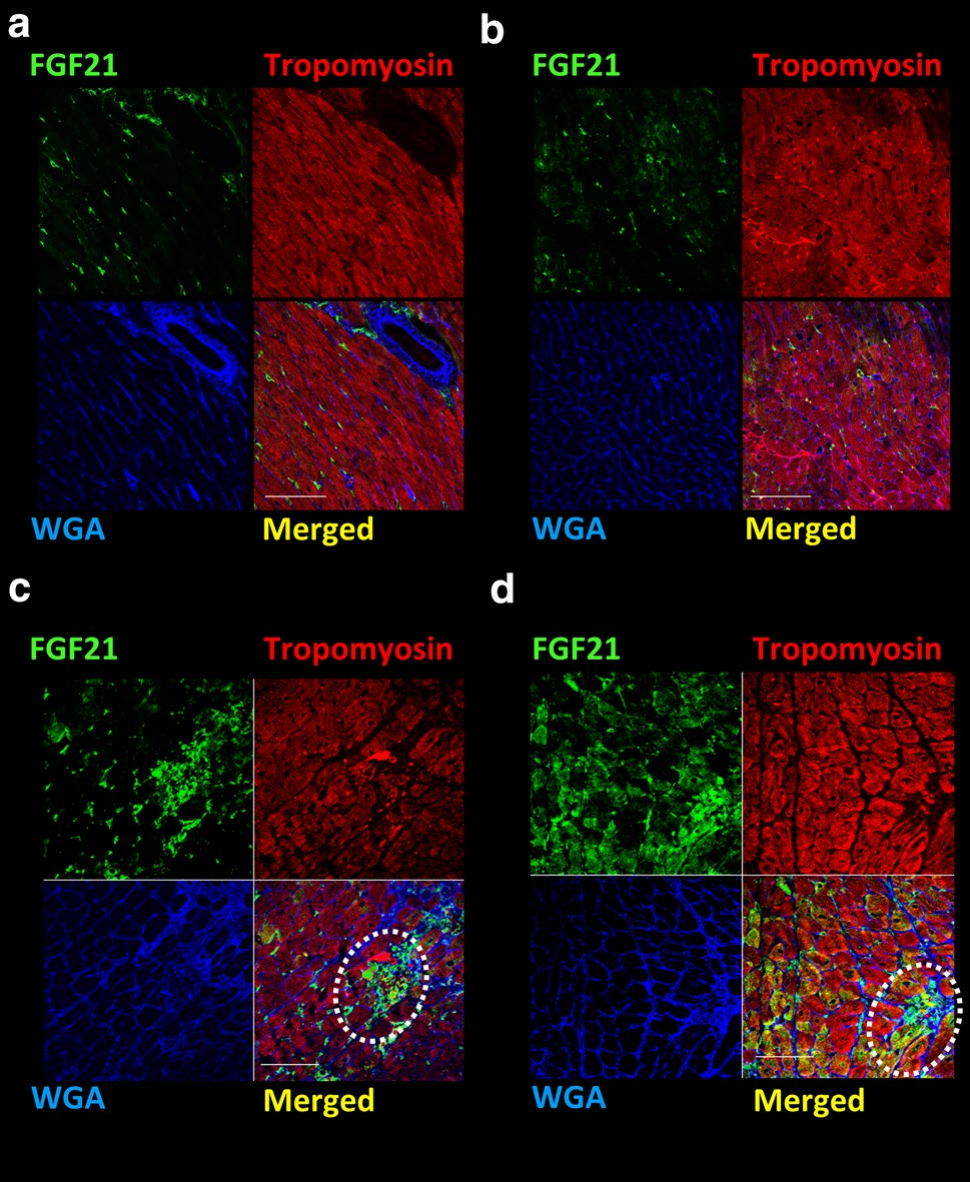
Fluorescent immunostaining of heart sections from 3-week TAC mice. Representative fluorescent immunostaining images. Vehicle-treated sham-operated (**a**), *vilda*-treated sham-operated (**b**). Vehicle-treated TAC-operated (**c**) and *vilda*-treated TAC-operated (**d**). FGF21 (green), Tropomyosin (myocytes) (red), and WGA staining (membrane/extracellular matrix) (blue). Dotted circles indicate non-myocyte positive staining for FGF21. Scale bars: 50 µm

Immunostaining revealed that FGF21 protein expression was increased in cardiac tissues following TAC (Fig. 4). FGF21-positive cardiomyocytes and stromal cells were observed in the area of interstitial fibrosis, which included cardiac fibroblasts and capillary vessels (Fig. 4c, d). *Vilda* treatment increased FGF21-positive cells in both cardiomyocytes and stromal cells (Fig. 4d).

### Vildagliptin treatment markedly increased FGF21 expression in human cardiac fibroblasts

**Fig. 5:**
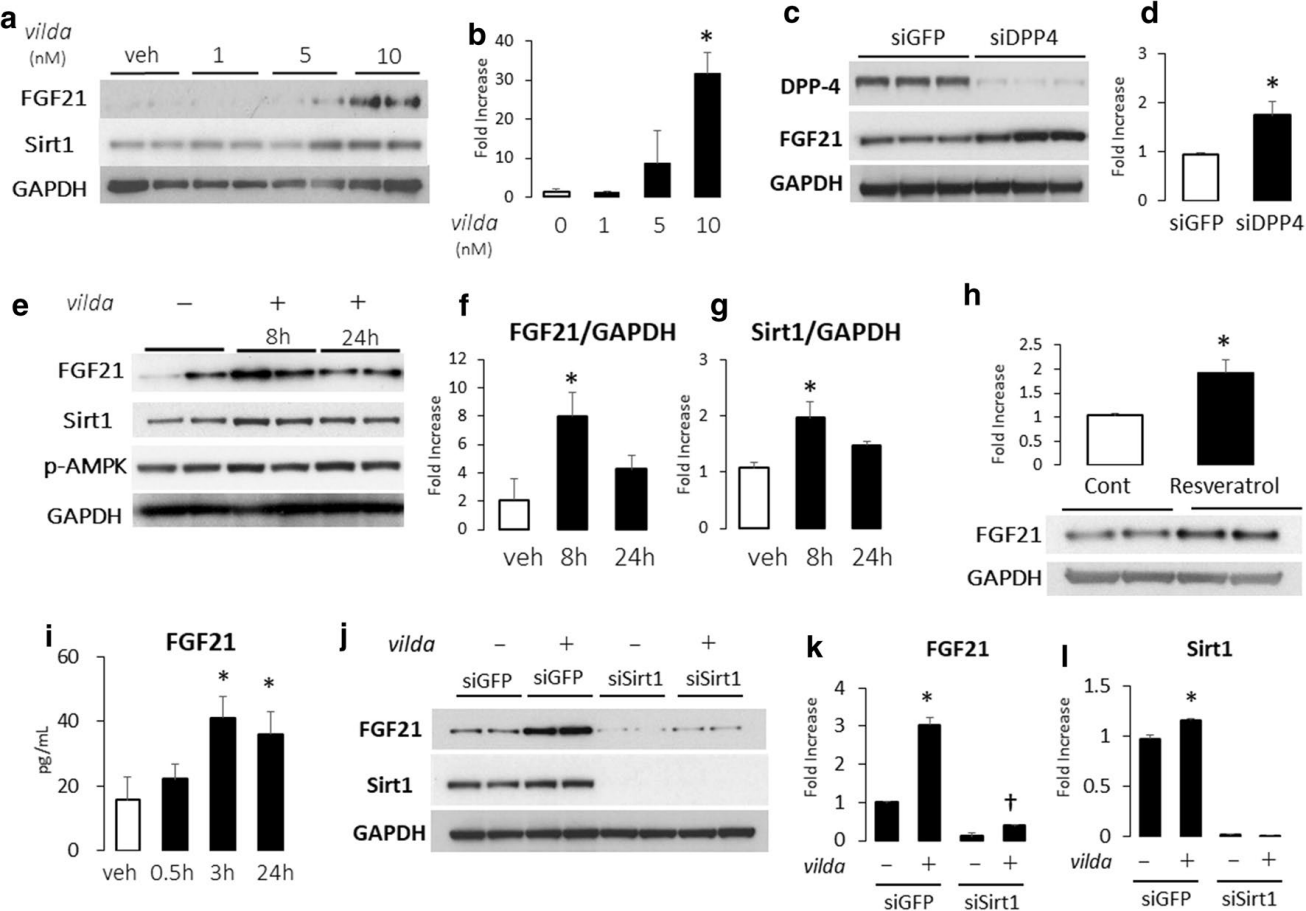
*Vilda* induces FGF21 expression through the Sirt1 pathway in human cardiac fibroblasts (HCF). **a** Western blot analysis of the effect of *vilda* treatment on FGF21 protein levels in HCF. Summarized data for FGF21 are shown in **b** (*N* = 4 for each experiment). Statistical analysis was performed unpaired two-tailed Student’s *t* test with Welch’s correction. *P < 0.05 vs 0 nM. **c** Gene silencing effect of DPP4 induced by siDPP4 transfection on FGF21 expression in HCF. Summarized data for FGF21 are shown in **d** (*N* = 3 for each experiment). Statistical analysis was performed unpaired two-tailed Student’s *t* test with Welch’s correction. *P < 0.05 vs siGFP. **e**–**g** Time course after *vilda* stimulation (10 nM) in HCF. A representative western blot analysis of FGF21, Sirt1, and P-AMPK protein expression in HCF treated with *vilda* for 8 or 24 h (**e**). **f**, **g** are summarized data for FGF21 (**f**) and Sirt1 (**g**) (*N* = 4 for each experiment). Sirt1 activator (Resveratrol: 50 nM) induced FGF21 protein expression in cell lysates of HCFs (**h**). Statistical analysis was performed unpaired two-tailed Student’s *t* test with Welch’s correction. *P < 0.05 vs vehicle. ELISA for FGF21 in medium supernatant of HCF stimulated with Resveratrol (50 nM) for 0.5, 3 or 24 h (**i**). Open bar, Veh-treated; closed bar, Resveratrol-treated. Statistical analysis was performed unpaired two-tailed Student’s *t* test with Welch’s correction. *N* = 4 for each experiment group. *P < 0.05 vs veh. **j**–**l** Gene silencing effect for Sirt1 in vilda-induced FGF21 expression. Western blot analysis of the effects of siSirt1 treatment on FGF21 expression in HCFs in the absence and presence of *vilda* (10 nM) (**j**). **k**, **l** Summarized data for FGF21 (**k**) and Sirt1 (**l**). *N* = 3 for each experiment. *P < 0.01 vs veh. ^†^*P* < 0.05 for effect from Sirt1 silencing on *vilda* response, two-way ANOVA

We next attempted to identify which cells exhibited *vilda*-induced FGF21 expression in vitro. In cultured neonatal rat cardiomyocytes, FGF21 mRNA expression was barely detected (data not shown). Hence, we focused on human cardiac fibroblasts (HCF). Interestingly, *vilda* dose-dependently increased FGF21 expression at the level of protein (Fig. 5a, b) in cultured HCF. Gene silencing induced by DPP4 siRNA (siDPP4) produced a significant increase in FGF21 expression in HCF (Fig. 5c, d). Figure 5e, f shows the time course of FGF21 induction by *vilda* treatment in HCF. The FGF21 increase was similar to the Sirt1 expression pattern (Fig. 5g). The Sirt1 activator Resveratrol increased FGF21 expression in HCF (Fig. 5h) and FGF21 secretion in culture medium of HCF (Fig. 5i), suggesting that Sirt1 promotes FGF21 expression in HCF. Gene silencing induced by Sirt1 siRNA (siSirt1) markedly decreased FGF21 expression and blunted *vilda*-mediated FGF21 induction (Fig. 5g–l).

## Discussion

We showed cardioprotective effects of the DPP4 inhibitor *vilda* in pressure-overloaded mouse heart induced by TAC. *Vilda* blocked cardiac hypertrophy and dysfunction, especially left-ventricular contractile efficiency. Myocardial glucose and fatty acid uptake were both improved by *vilda* administration following TAC. FGF21 is a potent regulator of energy metabolism and was up-regulated by *vilda* in the TAC heart. Immunostaining showed non-myocyte FGF21 production in the stressed heart. In cultured cardiac fibroblasts, *vilda* regulated FGF21 expression through the Sirt1 pathway. Given that FGF21 is a potent metabolic regulator, our study suggests two important concepts: (1) cardiac fibroblasts may regulate cardiac energy metabolism through FGF21 expression, and (2) the beneficial effects of the DPP4 inhibitor in the stressed heart may be mediated by the Sirt1–FGF21 pathway.

FGF21 is a peptide hormone that regulates energy homeostasis in several organs including the heart [13]. We and other groups previously showed that circulating FGF21 levels were increased in patients with cardiovascular disease [24, 25, 29]. FGF21 knockout mice have shown to exacerbate isoproterenol-induced cardiac hypertrophy and failure [26]. FGF21 knockout mice also show decreased fatty acid oxidation in the heart [26]. Glucose uptake is also enhanced by FGF21 in vivo [23, 30, 31]. These results suggest that FGF21 has a protective effect on pathological cardiac remodeling through metabolic regulation. Since the DPP4 inhibitor improved cardiac contractile efficiency and glucose/fatty acid uptake in pressure-overloaded heart, we focused on FGF21, especially in cardiac fibroblasts. Interestingly, in vitro stimulation with *vilda* markedly increased FGF21 expression and production in cultured HCF. However, *vilda*-induced FGF21 expression was not observed in cultured cardiomyocytes (data not shown). Interestingly, when DPP4 was silenced by siRNA in cultured HCF, FGF21 expression was increased (Fig. 5c, d), suggesting that the effect of Vilda on FGF21 was not due to any action other than DPP4 inhibition. It has been reported that stromal cell-derived factor 1a (SDF-1) and neuropeptide-Y (NPY), substrates of DPP4, activate cardiac fibroblast [32, 33], but we failed to show SDF-1- or NPY-mediated FGF21 induction in HCF (data not shown).

Several pathways to regulate FGF21 expression have been previously reported including Sirt1, AMPK, and FoxO3a [27, 28]. Phosphorylation of FoxO3a, which has been shown to be Akt-phosphorylation site and mediate anti-apoptotic effects [34], was increased by TAC but not significantly increased by vilda treatment (Fig. 3b, f). Our data showed that *vilda*-induced FGF21 expression in cardiac fibroblasts was Sirt1-dependent. Sirt1, a sirtuin family protein, is a cytoplasmic sirtuin that plays a protective role in cell death and oxidative stress [35]. In the heart, Sirt1 can modulate fatty acid oxidation, cardiac hypertrophy, apoptosis, oxidative stress, and autophagy [35]. We previously showed that recombinant FGF21 stimulates several metabolic signaling molecules related to mitochondrial homeostasis and cardioprotection including PGC1a, AMPK, and Sirt1, and feed-forward regulation of AMPK–Sirt1–FGF21 [24]. Our study indicates that fibroblast–myocyte interaction through FGF21 may regulate cardiomyocyte function. DPP4 inhibitor treatment promotes the interaction in stressed myocardium, resulting in a protective effect for cardiac energy metabolism.

**Fig. 6:**
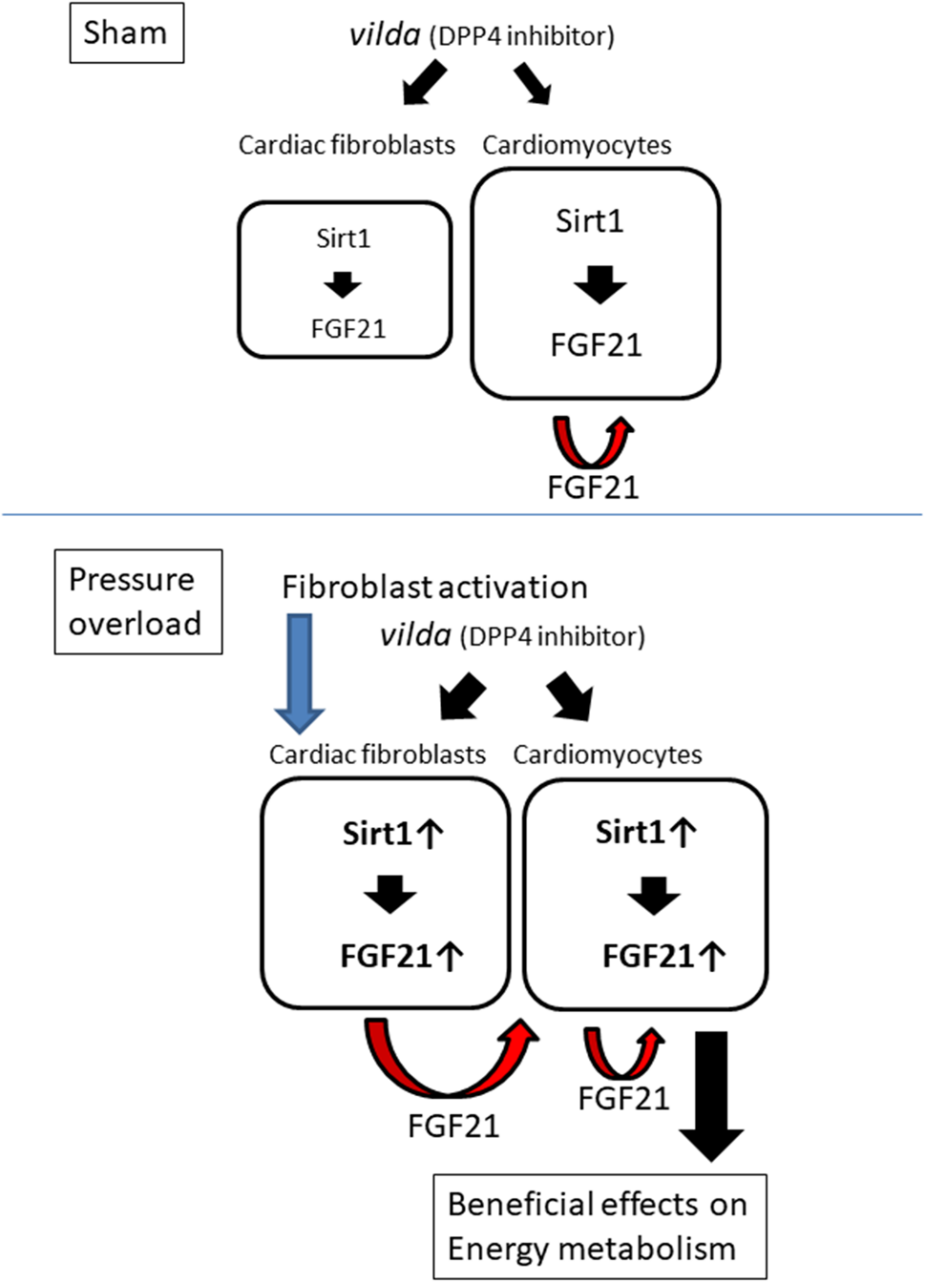
A schematic illustrating the Sirt1–FGF21 axis and *vilda*-mediated cardiac protection. *Vilda*-induced FGF21 expression in cardiac fibroblasts via Sirt1 and increased myocardial energy metabolism through fibroblast–myocyte interaction. Pressure overload induces cardiac-fibroblast activation in the heart, resulting in the fibroblast-mediated FGF21 pathway dominating (lower). Unknown inhibitory pathway(s) for FGF21 activation in activated cardiac fibroblasts is proposed to account for this observation

Cardiac fibroblasts are activated in the development of pathological cardiac remodeling induced by pressure overload, ischemia, neurohumoral activation, and inflammation [36, 37]. Activated cardiac fibroblasts contribute to extracellular matrix deposition as a healing response, whereas myocardial fibrosis can reduce cardiac function [36]. Fibroblast–myocyte interactions in the stressed heart have been shown to be involved in several remodeling processes, such as hypertrophy, angiogenesis, and fibrosis, through several secreted factors [16, 38]. Our findings present a new concept, in which cardiac fibroblasts can regulate myocardial energy metabolism through FGF21. Interestingly, in our cultured cell experiments, *vilda* induced FGF21 in cardiac fibroblasts, but the induction was not observed in cardiomyocytes. Given that activation of cardiac fibroblasts occurs only under pathological conditions in the adult heart, fibroblast-mediated myocardial energy regulation may provide an additional “safeguard” in heart pathophysiology. Figure 6 is a scheme illustrating the Sirt1–FGF21 axis and *Vilda*-mediated cardiac protection. *Vilda* induced FGF21 expression in cardiac fibroblasts via Sirt1 and increased myocardial energy metabolism through fibroblast–myocyte interactions. Since pressure overload induced cardiac-fibroblast activation in the heart, the fibroblast-mediated FGF21 pathway would be dominant.

It has been shown that peroxisome proliferator-activated receptor alpha (PPARα) regulates FGF21 expression and a PPARα agonist increases circulation FGF21 level [39]. We did not examine PPARα pathway in our model. Previous reports showed that PPARα acts as a cofactor of Sirt1 and may repress mitochondrial gene transcription, leading to heart failure [40]. Further study would be required to figure out the interaction of these two transcription factors on FGF21 regulation and cardiac function.

Unfortunately, we could not identify substrates of DPP4 that exert Sirt1–FGF21 activation in heart. In a recent study using endothelial cells, *vilda* has been shown to directly bind to transient receptor potential channel vanilloid (TRPV) 4 and activate Sirt1 pathway through substrate-independent fashion in endothelial cells [41]. TRPV4 channel is a calcium channel and activated by various physiological and pathological stimuli in various organs. Since TRPV4-mediated signaling is involved myocardial remodeling and fibrosis [42], further experiments would be required to figure out the role of TRPV4 and *vilda* in cardiac fibroblasts.

In a point of view of clinical setting, the dose what we used (50 mg/kg) was high. In previous basic studies using murine model, *vilda* was administered 5–50 mg/kg by oral gavage [14, 15]. We chose higher dose treatment to clarify pleiotropic effect of this drug in addition to antidiabetic effect. FGF21 does reduce body weight in obese model [43], but our model did not show the body weight change in the treatment group even in sham-operated mice. Since both FGF21 and *vilda* treatment could affect in glucose tolerance, additional experiments would be needed to examine insulin sensitivity and glucose tolerance for myocardial energy metabolism [44, 45]. Myocardial FDG uptake was increased in *vilda*-treated heart with TAC but not statistically significant in comparison with vehicle-treated heart with TAC, suggesting that myocardial insulin sensitivity and glucose tolerance could not explain our results.

In conclusion, *vilda* induced FGF21 expression in cardiac fibroblasts via Sirt1 and increased myocardial fatty acid and glucose metabolism in pressure-overloaded heart. Fibroblast–myocyte interaction through the FGF21 pathway induced by the DPP4 inhibitor may contribute to these results.
